# Cataphoresis in the Removal of Vital Pulps Followed by Immediate Root Filling

**Published:** 1897-07

**Authors:** S. Eldred Gilbert

**Affiliations:** Philadelphia.


ARTICLE XIII.

### CATAPHORESIS IN THE REMOVAL OF VITAL PULPS FOLLOWED BY IMMEDIATE ROOT FILLING.

S. ELDRED GILBERT, D. D. S., PHILADELPHIA.

My method of procedure is as follows: The rubber-dam being applied to the tooth to be operated on, the loose debris is removed and cotton saturated with a 25 per cent solution of cocain is placed in the cavity; the electrode attached to the positive pole is now placed in direct contact with the cotton, where it must remain immovable till the pulp is anesthetized. the negative pole is either held in the patient's hand, or, what is better, applied to the cheek. The electrodes must remain stationary, as there will be a shock if the current is broken (whatever instrument is used). The indicator on the volt selector is gradually turned up till it reaches twenty-five volts, where it is allowed to remain till there is no sensation, when the current is broken. In turning on the current there should be no pain; the patient is told to give an indication just as the pain point is reached; it is allowed to remain till there is no sensation, then turned up as before, and so on till the desired point is attained. Should it be turned rapidly enough to give pain, turn it back a little, when the pain will immediately cease. The pulp being anesthetized it is now ready for removal, which will be without pain in almost every case, and with but the slightest feeling in others. When possible, removal with the broach is preferred, but in many cases this is absolutely impossible. These roots are opened by means of Gates-Glidden drills and sulfuric acid till the operation is nearly completed (neutralizing with bicarbonate of soda). For finishing I

prefer to use muriatic acid, then neutralize with Liq. soda chlorinate. (I find Dr. Barker's acid applier, root drier, and canal filler is very handy in these cases). With a cotton wound broach an antiseptic is introduced into the canal, following with chloro-stopping (temporary stopping dissolved in chloroform to a creamy thickness). Then finish by inserting a temporary stopping cone. This is made by rolling temporary stopping between the thumb and fingers; several of these points of various sizes are kept ready for use. The root being permanently filled, the crown is finished with temporary stopping and allowed to remain for two weeks, and then permanently filled, or, after the root canals are filled, a permanent filling may be placed in the crown at once. I have treated a great many teeth in this manner, and not one has ever given the slightest trouble. For molars the first application will allow of the removal of the pulp from the largest canal without pain, but it will be found that usually on testing the remaining roots they are sensitive. The root in which there is no sensation is treated as before described, and filled. Cotton saturated with the cocain is again placed in the cavity, the current applied, which it will be found can be increased very rapidly, and in about five minutes the remaining canals are ready to be operated on. For teeth with single canals, including time after the dam is in place, opening, filling, completing and the removing of the rubber-dam averages forty-five minutes, and for molars an hour and a half. Sometimes it takes much less and sometimes a little longer. My greatest satisfaction is that I have no after treatment, and that I can finish the tooth at one sitting, without pain, and know that there will be no trouble from soreness afterward.—*Welch's Monthly.*

